# Unraveling the potential of breath and sweat VOC capture devices for human disease detection: a systematic-like review of canine olfaction and GC-MS analysis

**DOI:** 10.3389/fchem.2023.1282450

**Published:** 2023-11-01

**Authors:** Laetitia Maidodou, Igor Clarot, Michelle Leemans, Isabelle Fromantin, Eric Marchioni, Damien Steyer

**Affiliations:** ^1^ Twistaroma, Illkirch Graffenstaden, France; ^2^ CITHEFOR, EA 3452, Université de Lorraine, Nancy, France; ^3^ DSA, IPHC UMR7178, Université de Strasbourg, Strasbourg, France; ^4^ Clinical Epidemiology and Ageing, IMRB—Paris Est Créteil University /Inserm U955, Créteil, France; ^5^ Wound Care and Research Unit, Curie Institute, Paris, France

**Keywords:** breath, sweat, VOC, canine olfaction, sampling devices, gas chromatography, mass spectrometry

## Abstract

The development of disease screening methods using biomedical detection dogs relies on the collection and analysis of body odors, particularly volatile organic compounds (VOCs) present in body fluids. To capture and analyze odors produced by the human body, numerous protocols and materials are used in forensics or medical studies. This paper provides an overview of sampling devices used to collect VOCs from sweat and exhaled air, for medical diagnostic purposes using canine olfaction and/or Gas Chromatography-Mass spectrometry (GC-MS). Canine olfaction and GC-MS are regarded as complementary tools, holding immense promise for detecting cancers and infectious diseases. However, existing literature lacks guidelines for selecting materials suitable for both canine olfaction and GC-MS. Hence, this review aims to address this gap and pave the way for efficient body odor sampling materials. The first section of the paper describes the materials utilized in training sniffing dogs, while the second section delves into the details of sampling devices and extraction techniques employed for exhaled air and sweat analysis using GC-MS. Finally, the paper proposes the development of an ideal sampling device tailored for detection purposes in the field of odorology. By bridging the knowledge gap, this study seeks to advance disease detection methodologies, harnessing the unique abilities of both dogs and GC-MS analysis in biomedical research.

## 1 Introduction

Human body odors are widely studied to develop non-invasive disease diagnosis methods. A plethora of reviews exist on the subject, encompassing biomedical detection dogs (Gordon et al., 2008; Moser and McCulloch, 2010a; Lippi and Cervellin, 2012a; Pirrone and Albertini, 2017a; Catala et al., 2019) and instrumental analysis (Ligor et al., 2008; Dormont et al., 2013; de Lacy Costello et al., 2014; Monteiro et al., 2014; Prada et al., 2014; Robinson et al., 2018; Lippi and Heaney, 2020; Chai and Chua, 2021; Sinclair et al., 2021). Additionally, alternative systems like electronic noses have emerged since the mid-1980s. These systems utilize sensors to detect disease-specific biomarkers, offering quick real-time preliminary diagnoses at a lower cost. One of their drawbacks is the lower level of sample discrimination (Wilson and Forse, 2023). Electronic nose systems are less portable than canine olfaction due to potential restrictions posed by power, weight, or space requirements (Wilson and Forse, 2023). Despite this, the use of the dog as a diagnostic tool has not yet been standardized nor validated by health organizations (Bauër et al., 2022a). Therefore, GC-MS is of great interest to identify diseases-specific VOCs, detected by dogs. It can be employed as a complementary tool to validate canine olfaction-based diagnosis methods and to understand how dogs discriminate sick from healthy humans.

The choice of the sampling material plays a critical role in training dogs to recognize a specific pattern of volatile biomarkers. To effectively present the material to the dog, it must be appropriately sized and contained, such as a cloth, cotton gauze, swab, or a facemask capable of absorbing VOCs. The detection of precise odors can be challenging due to the variability in samples and the environment. Therefore, selecting an appropriate material that can collect and diffuse odors efficiently can greatly facilitate the dog’s sniffing process, leading to more accurate results during detection exercises.

**FIGURE 1:**
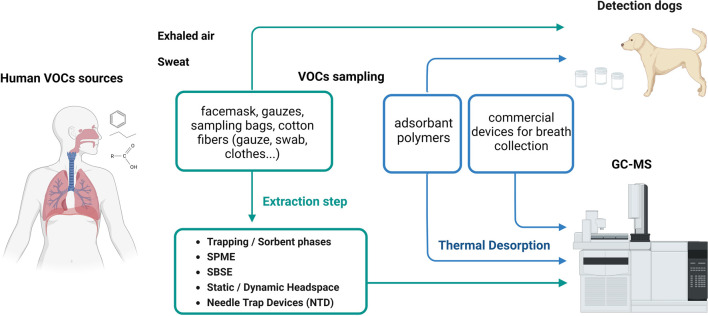
Flowchart (created in BioRender.com) illustrating the process for human VOCs sampling, extraction, and analysis by canine olfaction and GC-MS; GC-MS: Gas Chromatography coupled to Mass Spectrometry, SPME: Solid Phase Micro-Extraction, SBSE: Stir Bars Sorptive Extraction.

In the context of instrumental analysis using GC-MS for VOCs detection, it is essential to extract the VOCs from the sampling material and inject them into the analytical system. This paper focuses on discussing specific devices based on adsorbent polymers designed for training detection dogs. Among these devices, the most suitable ones are those that are both dog-compatible and can be directly used with GC-MS systems through thermal desorption, as illustrated in Figure 1.

In addition to the medical context, sniffing dogs find extensive application in forensics. Standardized protocols are used to train these dogs for explosives or illicit substance detection and criminal suspect identification (Lorenzo et al., 2003). Commonly, hands and feet serve as the VOCs sources for suspect identification, and gauze is often employed for sweat sampling (Cuzuel et al., 2017). Notably, The Scent Transfer Unit or STU100 (odor suction tool) plays a pivotal role in concentrating odors on gauze within this context (Curran et al., 2010a; Degreeff and Furton, 2011). This unit comprises a vacuum pump that actively samples VOCs emitted from any object onto a gauze.

The National Institute of Science and Technology (NIST) has developed a polymer-based adsorption canine training device made of polydimethylsiloxane (PDMS). This device, safe for the dog (non-toxic, non-infectious) (MacCrehan et al., 2020), has been employed in training dogs for explosive detection (MacCrehan et al., 2018a; Simon et al., 2021). Additionally, Getxent^®^ tubes (Biodesiv, France), consisting of a patented adsorbent polymer, have been developed for detection dog training and are utilized in forensics (Simon, 2022).

This comprehensive bibliographic study delves into a thorough examination of the methodologies utilized for comprehending diverse techniques of odor capture. Specifically, the study focuses on canine olfaction and/or Gas Chromatography-Mass Spectrometry (GC-MS) systems within the context of medical odor analysis. By delving into the intricacies of these methodologies, this study aims to shed light on the intricate processes involved in capturing odors, thereby contributing to the advancement of our understanding in the field.

## 2 Materials and methods

### 2.1 Literature search

In adherence to the widely recognized Preferred Reporting Items for Systematic Reviews and Meta-Analyses (PRISMA) standards, our search was conducted independently on both Pubmed and Web of Science. PRISMA is the most commonly utilized method for systematic reviews ((Moher et al., 2022).

#### 2.1.1 Canine olfaction

In this study, the research is focused on the different scent collection devices related to the analysis of gaseous non-invasive materials: exhaled air, and sweat/body odor, in a medical context. The subsequent sequence is researched in Web of Science and PubMed databases: *((dog)OR(canine)) AND ((odor)OR(odour)OR(smell)OR(sniff)OR(volatile organic compounds)OR(volatile)) AND ((breath)OR(exhaled air)OR(sweat)) AND ((diagnosis)OR(disease)OR(medical)) AND ((human)OR(patient)OR(subject))*. Studies have been selected without date restrictions until March 2023.

#### 2.1.2 GC-MS analysis

The subsequent sequence was researched in Web of Sciences and PubMed databases: *((GC-MS) OR (gas chromatography) OR (volatolomics)) AND ((odor) OR (odour) OR (volatile organic compounds) OR (volatile)) AND ((breath) OR (exhaled air) OR (sweat) OR (body odor)) AND ((diagnosis) OR (disease) OR (medical)) AND ((human) OR (patient) OR (subject))*. Studies have been selected without date restrictions until March 2023.

### 2.2 Study selection and eligibility criteria

**FIGURE 2:**
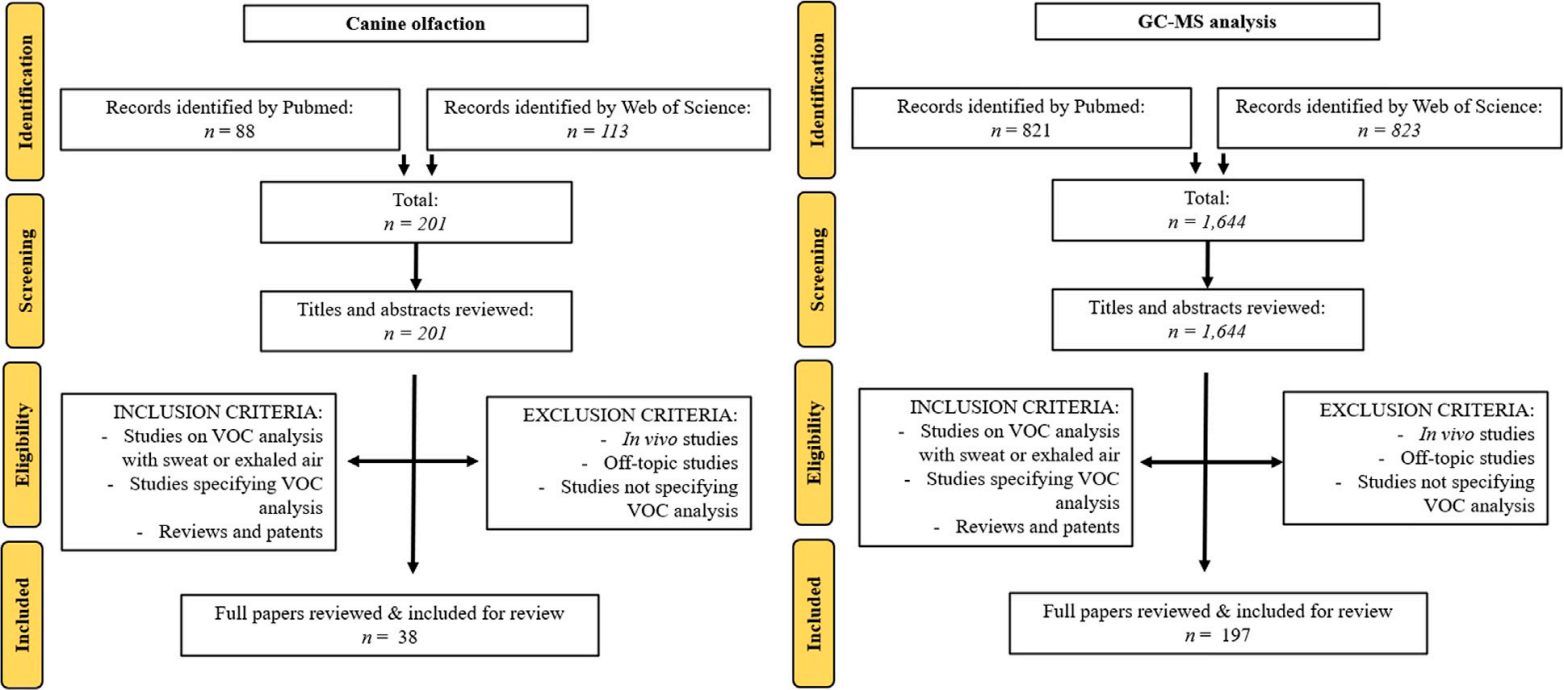
PRISMA flow-chart for canine olfaction and GC-MS analysis.

Following the PRISMA standards, data obtained from the independent searches on Pubmed and Web of Science were consolidated into a worksheet file, incorporating essential details such as DOI number, title, publication year, authors, journal, and abstracts. For canine olfaction, the initial search yielded a total of 201 records, with 88 from PubMed and 113 from Web of Science. After a comprehensive evaluation of 201 full-text papers, 163 were excluded based on relevance, resulting in the inclusion of 38 papers in the final systematic review. For GC-MS analysis, a total of 197 papers were included with initially 821 articles from Pubmed and 823 from Web of Science. After the removal of duplicates, the remaining titles and abstracts were carefully reviewed to identify studies relevant to the topic. To ensure thoroughness, two researchers (L.M. and D.S.) independently assessed the studies, resolving any discrepancies through discussion. Articles were considered if they featured a dedicated “material and methods” section that comprehensively delineated the protocol employed for sampling VOCs. A prerequisite was the presence of at least one sampling material being explicitly cited. Furthermore, the study took into account reviews and patents. Importantly, no differentiation was made based on the level of pathologies, ensuring a broad and inclusive scope. Conversely, articles were excluded if they lacked pertinent information concerning VOCs sampling. Additionally, no *in vivo* studies were included (Figure 2). To ensure a comprehensive overview, reference lists of the included articles were also screened based on their titles and abstracts, enabling the inclusion of further pertinent articles.

### 2.3 Data extraction

The relevant data were extracted from the selected studies. Standardized tables were designed to abstract the studies of interest. Table 1 highlights instances where PDMS-based sampling devices are employed to capture volatile compounds for medical odor analysis. Table 2 showcases practical scenarios demonstrating the use of fabric-based techniques to capture volatile compounds, contributing to odor analysis. In Table 3, various extraction techniques post-sample collection are illustrated, vital for isolating volatile compounds for subsequent analysis.

**TABLE 1:** Application examples of PDMS-based sampling devices.

Body area	Sampling device	Sampling method	Desorption method	Sample storage conditions	Analysis system	GC-column	Sensibility	Ref.
wrist and ankle	a 25 cm long tube made of medical grade PDMS (0.64 mm OD × 0.3 mm ID, Sil-Tec^®^).	passive sampling for 1 h	Thermal desorption at 250°C in a splitless mode for 30 s (PDMS sampler is inserted into a glass inlet liner (Agilent)	stored in aluminum foil at 4°C for 48 h	GCxGC-TOFMS	1D column: Rxi-5Sil (Restek) 30 m × 0.25 mm × 0.25 μm. 2D column: Rxi-17Sil (Restek) 1 m × 0.25 mm × 0.25 µm	not specified	Wooding et al. (2020)
forehead	PDMS patch (5 mm × 15 mm x 0.45 mm)	passive sampling for 30 min	Thermal desorption at 180°C for 10 min. Cryo-focusing at −10°C. Injection splitless at 300°C for 5 min	stored into TD-tubes at −80°C for 21 days	GC-MS	DB-5 MS (Agilent) 60 m × 0.25 mm x 0.25 µm	170 to 200 pg/cm	Martin et al. (2016)
forearms and abdomen	PDMS patches (20 mm × 15 mm × 0.45 mm)	passive sampling for 5–120 min	Thermal desorption at 180°C for 5 min. Cryo-focusing at −10°C. Injection splitless at 300°C for 3 min	stored into TD tubes at 4°C for 24 h	GC-MS	DB-5MS (Agilent) 60 m × 0.25 mm x 0.25 µm	50 pg to 100 ng per sample (relative estimation)	Riazanskaia et al. (2008)
upper back, forearm, and back thigh	PDMS round patches (6, 11 and 17 mm diameter, 0.25 mm, thickness)	passive sampling for 60 min	Thermal desorption at 250°C for 3 min. Cryo-focusing at −120°C. Injection splitless at 280°C	stored no longer than 72 h	GC-MS	RK13870 (Restek) 30 m × 0.32 mm x 1.8 µm	not specified	Jiang et al. (2013)
forearms	Twisters (10 mm,0.5 mm in film thickness, 24 µL PDMS phase volume, Gerstel)	active sampling (Twisters roll on the skin), the sampling time is not specified	Thermal desorption at 280°C for 10 min. Cryo-focusing at −60°C. Injection at 280°C for 10 min	stored at 4°C for 14 days	GC-MS	DB-5MS (Agilent) 20 m × 0.18 mm x 0.18 µm	not specified	Penn et al. (2007)
			Thermal desorption at 250°C for 3 min. Cryo-focusing at −80°C. Injection at 280°C for 10 min	stored at 4°C for 14 days	GC-MS	DB-5MS (Agilent) 20 m × 0.18 mm x 0.18 µm	not specified	Soini et al. (2006)
axilla			Thermal desorption at 250°C for 3 min. Cryo-focusing at −80°C. Injection at 280°C for 10 min	stored at 4°C for 20 days	GC-MS	DB-5MS (Agilent) 20 m × 0.18 mm x 0.18 µm	not specified	Xu et al. (2007)

**TABLE 2:** Application examples of sampling protocols using fabrics.

Body area	Sampling device	Sampling method	Extraction method	Adsorbent phase	Sample storage conditions	Analysis method	Ref.
hand	DUKAL brand, sterile, 2 × 2, 8ply, gauze sponges	active sampling in direct contact with the skin (palm hands)	SPME at room temperature for 21 h	CAR/DVB/PDMS	stored at room temperature for 24 h	GC-MS	Curran et al. (2010b)
feet	a strip of cotton wool (3.0 g)	sampling in direct contact with the feet skin (cotton placed in the socks) for 6 h	SPME 10 min at 55°C	polyacrylate (85 mm) 100-mm-long fiber	not specified	GC-MS	Caroprese et al. (2009)
genitourinary area	gauze made of cotton and cellulose, of 20 × 13 cm dimension	sampling in direct skin (genito-urinary area) overnight	Headspace 100°C for 30 min	n/a	not specified	GC-MS	Rodríguez-Esquivel et al. (2018)
palm of hands	gauze	sampling in direct contact with the skin (2 cm square area of the palm is wiped for 1 min with 0.1 g of dry gauze)	SPME at 50°C for 45 min	PDMS/DVB	stored 24 h at 4°C	GC-MS	Saito et al. (2021)
upper back	medical gauze	active sampling by swabbing the gauze on the skin to collect the sebum and the sweat (sampling time not specified)	DHS; incubation for 5 min at 60 °C, trapping by purging 500 mL of the sample headspace at 50 mL/min with dry nitrogen through an adsorbent tube kept at 40°C	Tenax TA	stored at −80°C in inert plastic bags	GC-MS	Trivedi et al. (2019b)
armpit	absorbent pads	passive sampling (pads are attached via stainless steel poppets in pre-cleaned T-shirts)	HSSE in 250 mL Scott-Duran GLS80 bottles with a PDMS stir bar (1 cm long, 1 mm thickness) at 60°C for 2 h	PDMS	stored in bags and vacuum-sealed at −28°C	GCxGC-TOFMS	Smeets et al. (2020)
hand	Sterile cotton gauze pads (100% cotton) Dukal		SPME; equilibration at 50°C for 24 h, extraction for 15 h	2 cm fiber, 50/30 µm DVB/CAR/PDMS	stored in cleaned 10 mL vials, sealed and secured with parafilm around the screw cap opening	GC-MS	Crespo-Cajigas et al. (2023)
palm of hands	Gauze pads were DUKAL brand, 100% cotton, sterile, 2 × 2, 8-ply, gauze sponges	Subjects hold the gauze between the palms of their hands as they walked outdoors for 10 min	SPME at room temperature for 21 h	50/30 µm DVB/CAR/PDMS	stored in a sealed 10 mL glass vial for 24 h prior to extraction at ambient temperature	GC-MS	Curran et al. (2007)
armpit	Gauze pads were Dukal brand, sterile, 2 × 2, 8-ply, gauze sponges	Subjects wiped a gauze on their armpit after 30 min of outdoor physical exercised	SPME at room temperature for 15 h	50/30 µm DVB/CAR/PDMS	stored in a sealed 10 mL glass vial for 24 h prior to extraction at ambient temperature	GC-MS	Curran et al. (2005b)
bust	cotton-shirts	passive sampling: subjects wear the shirt for 3 days; a rectangular piece 20 × 30 cm is cut and stored in a 10 L Tedlar^®^ bag	DHS at 23°C for 18 h, using a sampling pump at a flow rate of 1.8 L/min with purified air	TENAX-TA (GL Science)	stored in a 10 L Tedlar^®^ bag at room temperature in a dark place for no longer than 1 day	GC-MS	Haze et al. (2022).

**TABLE 3:** Application examples of extraction techniques after sample collection.

Source	Sampling device	Extraction method	Adsorbent phase	Protocol	Analysis system	Sensibility	Ref.
Breath	Tedlar^®^ bag, 1 L	NTD	PDMS, Carbopack X, Carboxen 1,000	air pump, 30 mL/min	GC-MS	ppb	Monedeiro et al. (2021)
	sampling bag		Carbotrap-B, Carbopack-X	air pump, 30 mL/min	GC-ToF-MS	ppb	Pizzini et al. (2018)
	sampling bag	SPME	Carboxen, PDMS	25 min at room temperature	GC-MS	ppt	Koureas et al. (2020)
	sampling bag		DVB, Carboxen, PDMS	60 min at 22°C	GCxGC-ToF-MS	ppq (pg/L)	Caldeira et al. (2012)
	Tedlar^®^ bag, 3 L		Carboxen, PDMS	10 min at 40°C	GC-MS	ppb	Mochalski et al. (2014)
	sampling bag	TF-SPME	PDMS Thin film, 5% Carboxen	3 h at 25°C	GC-MS	ppb	Murtada et al. (2021)
	FlexFilm bag, 3 L	Trapping in TD tube	Tenax, Carbograph, Carboxen	air pump, 200 mL/min	GCxGC	ppq (pg/L)	Berna et al. (2021)
					BenchTOF-MS		
	sampling bag		Tenax	air pump, 250 mL/min	GC-MS	ppb	Brinkman et al. (2017)
	sampling bag		Tenax TA	air pump, 100 mL/min	GC-MS	ppb	Broza et al. (2017)
Sweat	gauze	SPME	PDMS/DVB	45 min at 50°C	GC-MS	LOD for 2-nonenal: 2.4 pg/cm^2^/h	Saito et al. (2021)
	cotton-shirts	DHS	TENAX-TA (GL Science)	at 23°C for 18 h, using a sampling pump at a flow rate of 1.8 L/min with purified air	GC-MS	ppm (concentrations in ng per mg skin surface lipids)	Haze et al. (2022)

In the Supplementary Table S1, a detailed breakdown of sweat analysis using Gas Chromatography-Mass Spectrometry (GC-MS) is provided. Supplementary Table S2 outlines diverse extraction methods for Gas Chromatography-Mass Spectrometry (GC-MS) analysis of breath samples collected. Supplementary Table S3 offers a comprehensive comparison of odor capture systems, applicable to canine olfaction studies and Gas Chromatography-Mass Spectrometry (GC-MS) analysis.

## 3 Results

### 3.1 Canine olfaction

In 1989, a first study suggested the potential of dogs to diagnose cancer (Williams and Pembroke, 1989). Over the next 2 decades, the interest in this area grew exponentially, as evidenced by Moser *et al.*’s review in 2010, which identified 531 publications on cancer detection by dogs (Moser and McCulloch, 2010a). Among the various reviews (Moser and McCulloch, 2010b; Lippi and Cervellin, 2012b; Guest et al., 2019; Jendrny et al., 2021a), Jendrny *et al.*’s recent review (Jendrny et al., 2021a) stands out as it followed strict selection criteria including peer-reviewed studies with reported detection rate and the diagnostic accuracy (sensitivity/specificity) of the diseases under investigation. From the pool of studies, they focused on studies, with a substantial overlap between the selection made by Pirrone *et al.* (Pirrone and Albertini, 2017b) (17 selected publications) and by Moser *et al.* (Moser and McCulloch, 2010a) (6 publications retained out of 531). Two key aspects were highlighted in the selected studies: the diseases studied and sampling sources. Notably, odor detection by dogs was predominantly explored for hypoglycemia (25%), epilepsy (10%), and prostate cancer (10%). The primary non-invasive matrices studied included urine (20% of publications), sweat (10%), breath and sweat together (10%), and feces (10%) (Jendrny et al., 2021b). However, this review does not delve into the VOCs sampling from urine, leaving it to be explored in future studies.

#### 3.1.1 Human sweat capture for disease detection by dogs

In the realm of disease detection, an intriguing focus emerges in Section 3.1.1, where we delve into the captivating domain of human sweat capture for canine olfaction. In the case of malaria diagnosis by detection dogs, researchers conducted a randomized and blinded study where children wore socks overnight before being presented to the dogs (Guest et al., 2019). Encouraging results were obtained from the analysis of 175 samples, with detection dogs showing a sensitivity of 72% and a specificity of 91%. For hypoglycemia detection, sniffing dogs are trained (1–2 weeks) to assist diabetic patients by sniffing their blood glucose status without the need for any specific sampling device (Gonder-Frederick et al., 2017; Rooney et al., 2019). However, in two studies investigating blood glucose level recognition in type I diabetes by dogs, sweat was collected on gauze from the arms (Dehlinger et al., 2013) or neck (Hardin et al., 2015) and stored (the storage time is not mentioned) at −18°C before being analyzed by the dogs (Hardin et al., 2015). So far, the results obtained using hypoglycemia sniffing dogs are not as accurate as standard invasive methods for blood glucose determination (ISO15197:2003 standards prescribe a maximal deviation of 20% in 95% of measurements when blood glucose levels are above 75 mg/dL and a deviation of 15 mg/dL when below 75 mg/dL (Eerdeken et al., 2020)). The sensitivity of hypoglycemia sniffing dogs ranged from 50% to 87.5%, and the specificity from 89.6% to 97.9% (Hardin et al., 2015).

In the context of epilepsy diagnosis, a study by Catala *et al.* (Catala et al., 2019) used a similar protocol to Hardin *et al.* (Hardin et al., 2015), where patients rubbed gauze on their necks and placed it in a zip lock bag (Catala et al., 2019). Dogs detected epileptic with a sensitivity of 87% and a specificity of 98% in this small cohort study (5 patients). Another epilepsy-related study used gauze to collect sweat from different body parts before storing it in an inert sampling bag (Mylar^®^) and presenting it to the sniffing dogs (Maa et al., 2021). In this case, dogs were able to alert the subject before a seizure happened with a probability of 82%.

Several studies focused on the detection of COVID-19 by canine olfaction, including the work of Devillier *et al.* (Grandjean et al., 2020; Devillier et al., 2022). These studies used gauze (no brand name specified, 20 mins of contact to recover up to 76 mg of sweat) and Getxent^®^ tubes to collect sweat from underarms, and the sampling devices were stored at 18°C and 6°C in separate laboratories before being presented to the dogs. Lately, Jendrny *et al.* conducted a review of 22 studies related to SARS-CoV-2 detection by canine olfaction (Jendrny et al., 2021b), with sweat being the most commonly used VOCs source (11 of 22 studies). Dogs’ performances in terms of sensitivity range from 65% to 100% and in specificity from 76% to 99% in these studies (Jendrny et al., 2021b).

Furthermore, one study evaluates the effect of freezing sweat samples collected on gauze between collection and usage (Lenochova et al., 2009), with human panelists evaluating the odors. Interestingly, no differences were observed between fresh and 6 months frozen samples.

#### 3.1.2 Human exhaled air capture for disease detection by dogs

Several systems have been developed to capture VOCs from exhaled air and present them to dogs for evaluation. However, there is currently no standardized method, and different teams employ their approaches to collect odorant compounds. Two main methods are commonly used for collecting odors for dog evaluation: breath sampling tubes and sampling bags.

Breath sampling tubes, often custom-made, are used in some studies on the analysis of exhaled air. These tubes can range from 15 cm tubes (Montes et al., 2017) to 20 cm long (Reeve et al., 2020) and are equipped with materials like cotton balls or two layers of glass wool (one hydrophobic and hydrophilic) to trap the breath samples (McCulloch et al., 2006; Ehmann et al., 2012; Walczak et al., 2012; Montes et al., 2017).

Sampling bags are another approach used to collect exhaled air for dog evaluation. In this method, subjects exhale air into special bags, which are then presented to the dogs for the detection of various conditions, such as colorectal cancer (Sonoda et al., 2011).

More recently, researchers have explored the use of surgical facemasks for COVID-19 detection through canine olfaction (Devillier et al., 2022; Mendel et al., 2021). Mendel *et al.* (Mendel et al., 2021) pre-tested patients at a healthcare facility for COVID-19 and then asked to wear masks for 30–45 mins. These facemasks were then collected in special bags and transported to the testing laboratory. Masks were exposed to UV light to inactivate the potential virus particles. UV treatment of the facemasks has been tested and does not affect the nature of VOCs (Martin et al., 2020). After, the facemasks were cut into small squares for presentation to the dogs for sniffing tests and instrumental analysis. The study demonstrated promising results, with canine olfaction achieving an accuracy greater than 90% ((Mendel et al., 2021).

However, it is worth noting that using surgical facemasks for breath sampling may introduce additional VOCs from sweat and sebum present on the patient’s facial skin. Despite, this potential confounding factor, the study achieved impressive sensitivity and specificity values after 1 month of training dogs for COVID-19 detection (Mendel et al., 2021; Devillier et al., 2022).

Regarding storage of breath samples, different studies have employed varying temperatures, such as in cold storage at 4°C (Sonoda et al., 2011; Devillier et al., 2022) or at room temperature (McCulloch et al., 2006; Ehmann et al., 2012; Walczak et al., 2012; Reeve et al., 2018; Reeve et al., 2020), for varying lengths of time, which strongly depend on the study length. However, justification for these specific storage conditions is not always provided in the literature.

### 3.2 GC-MS analysis

The analysis of human sweat and breath is highly diverse and varies significantly among different research teams. Each team tends to use distinct odor sampling systems tailored to the specific objectives of their studies. In the subsequent section, we will delve into the description of sweat sampling devices concerning disease diagnosis through GC-MS analysis.

#### 3.2.1 Sweat sampling devices for GC-MS analysis

##### 3.2.1.1 Use of PDMS for body odor sampling

Several devices based on polydimethylsiloxane (PDMS) have been developed to collect sweat from human skin for analysis (Soini et al., 2006; Bicchi et al., 2007; Xu et al., 2007; Riazanskaia et al., 2008; Sgorbini et al., 2010; Jiang et al., 2013; Martin et al., 2016; Roodt et al., 2018; Wooding et al., 2020; Wooding et al., 2021). These Medical-grade PDMS-based materials are often fashioned into small pieces known as “patches” or “skin patches”, which can be placed in direct contact with the skin to allow passive sampling. After sweat collection, these patches are subjected to a thermal desorption process to desorb VOCs. The analytes are then cryo-focused into a cold trap before being injected into the gas chromatography column in a splitless mode for further analysis (details are provided in Table 1).

Researchers choose the size and shape of the PDMS-based skin patch based on the sampling area and specific study requirements. For example, Martin *et al.* (Martin et al., 2016) used rectangular patches measuring 5 mm × 15 mm patches with a thickness of 0.45 mm, to collect sebum and sweat from the whole forehead of volunteers. Round patches of various diameters (6,11, and 17 mm) and a thickness of 0.25 mm were employed to collect sweat from the upper back, forearm, and back tight (Jiang et al., 2013). The signal intensity obtained through TD-GC-MS increases with the size of the patch (Jiang et al., 2013), indicating its influence on sensitivity. Additionally, Wooding *et al.* (Wooding et al., 2020) utilized PDMS patches to sample VOCs from the arm and abdomen skin surface for durations ranging from 5 to 120 mins. The results demonstrated a sensitivity increase proportional to the sampling time for most compounds, except for highly volatile ones like 2,4,6-trimethylcarbazole.

In some cases, to prevent the PDMS patch from getting impregnated with skin sweat and sebum, it is placed in a "sandwich” between two stainless steel meshes (Jiang et al., 2013), which additionally aids in reducing background noise. For certain research involving mosquito attractants emitted from the skin of healthy subjects, PDMS-based samplers in the form of bracelets and wristbands have been developed (Wooding et al., 2020). These samplers are made of medical-grade PDMS tubing and are enclosed by Mylar^®^ sheeting (Hydroponic) to avoid contamination from surrounding air. It is recommended to store PDMS-based samplers at 4°C (Riazanskaia et al., 2008; Wooding et al., 2020) for no longer than 24 h (Riazanskaia et al., 2008).

PDMS-based Twisters, commonly used for Stir Bars Sorptive Extraction (SBSE), have also been employed as sampling materials to collect VOCs from skin regions like the arms (Soini et al., 2006; Penn et al., 2007; Xu et al., 2007) and axilla (Xu et al., 2007). These twisters can be rolled on specific areas of the skin, enabling researchers to identify individuals and gender-volatile markers in human body odor (Penn et al., 2007). About 400 compounds are detected and 100 are identified. Twisters were stored for up to 14 or 20 days at 4°C before analysis (Penn et al., 2007; Xu et al., 2007).

PDMS-based samplers offer numerous advantages, including ease of use and reproducibility in the sampling procedure, self-administrability, and portability. These devices can be stored in empty stainless steel thermal desorption tubes, making transportation and storage convenient. Moreover, the size and shape of the patch can be customized for different applications, making them suitable for canine olfaction. In forensics, PDMS has shown relevance in capturing and releasing explosives’ odorants for dog sniffing applications (MacCrehan et al., 2018b).

While quantification of VOCs in sweat is infrequently reported. Two studies (Riazanskaia et al., 2008; Martin et al., 2016), shown in Table 1, utilized PDMS skin patches and provided detected and identified VOCs concentration range values.

Overall, PDMS-based sweat sampling devices offer a valuable and versatile tool for capturing volatile compounds from the skin, with potential applications in various scientific fields and canine olfaction studies.

##### 3.2.1.2 Use of Sorbstar^®^ tubes for body odors sampling

In the thesis work of V. Cuzuel, Sorbstar^®^ tubes demonstrated good efficiency in capturing and releasing VOCs from the palms of hands (Cuzuel, 2022). This sampling approach is akin to SBSE, and active sampling can be holding and rolling the Sorbstar^®^ tubes in the palms of the hands. Despite its promising performance, Sorbstar^®^ tubes are not widely utilized in research studies, as only one paper implementing this sampling device has been identified.

##### 3.2.1.3 Use of gauze, clothes, or cotton for body odors sampling

In numerous experimental studies, gauzes have been utilized for body odor sampling (Curran et al., 2005a; Curran et al., 2010b; Rodríguez-Esquivel et al., 2018; Crespo-Cajigas et al., 2023) (protocols detailed in Table 2). However, the brand and type of gauze used are often not explicitly specified. A comprehensive comparison of nine commercially available gauzes (Dukal, J&J, Nexcare, IMCO, Eckerds, Cotton Roll, King’s Cotton, Polish Absorbers, and Hungarian Cotton) was conducted by Furton and Curran in 2006, as published in a patent (The Patent Cooperation Treaty, 2007). Among these gauzes, Nexcare was found to release the highest number of volatile compounds (58 VOCs), while Dukal released the least VOCs (12 VOCs). This variation implies that the choice of gauze can significantly influence GC-MS analysis and dog sniffing results. Proper cleaning of the gauze before sweat sampling is crucial to ensure accurate and reliable sampling. The patent suggests cleaning vials and septa with acetone and then heating them at 210°C for 48 h to remove any residual VOCs traces before SPME GC-MS analysis ((The Patent Cooperation Treaty, 2007)).

In the context of studying the primary odor of subjects and differentiating between volunteers based on their body odor, gauzes have been used (Curran et al., 2010b). A protocol involving a pump and a supercritical fluid extractor is employed to clean the gauze before sweat sampling (Curran et al., 2010b). Cleaned gauze were placed on the subject’s hands for 5 mins following a hand-washing protocol and then stored at room temperature for 24 h. VOCs are subsequently extracted using SPME for an extended duration (21 h) at room temperature and analyzed by GC-MS. This study, conducted in 2009, provided the first evidence of the possibility of individual discrimination based on the analysis of VOCs from hands (Curran et al., 2010b).

Trivedi *et al.* (Trivedi et al., 2019a) conducted a study prompted by the discovery of a "super smeller” named Joy Milne who detects a distinct odor in her Parkinson’s disease-afflicted husband. They investigated healthy and Parkinson’s disease patients, totaling 64 subjects. Sweat and sebum were collected from their upper backs using gauze which were then stored at −80°C for analysis. The researchers employed DHS (Dynamic Headspace) extraction to extract VOCs from the gauze, followed by GC-MS coupled with an olfactometer. Results revealed that Perillic aldehyde tended to decrease for Parkinson’s disease patients, while three other components (Hippuric acid, Eicosan, and Octadecanal) increased. The peer-reviewed publication, originally an application note (Anatune, 2019), presented a solution to analyze the volatile compounds captured on gauze. In a subsequent study in 2021 (Sinclair et al., 2021) the research team failed to replicate the same biomarkers as in their 2019 study (Trivedi et al., 2019a).

Haze *et al.* (Haze et al., 2022) conducted a study on the evolution of t-2-nonenal during aging, utilizing T-shirts as sampling devices. Participants wore the T-shirt for 3 days, and a portion of the cloth was placed in a 10 L Tedlar^®^ bag. VOCs were extracted using a sampling pump to mimic dynamic headspace extraction (DHS), with a constant flow rate of 1.8 L/min. To maintain the 10 L capacity in the bag, deodorized air was supplied, and DHS collection was performed at 23°C for 18 h. VOCs were trapped into a Tenax^®^ TA cartridge and later subjected to liquid desorption using 10 mL of diethyl ether. In contrast, results from an American study contradicted these findings, suggesting that trans-2-nonenal is not a volatile biomarker of aging in a non-Japanese population (Gallagher et al., 2008).

More details on analytical methods, including desorption parameters, GC-MS system, and chromatographic columns are provided in Supplementary Table S1.

#### 3.2.2 Exhaled air sampling devices for GC-MS analysis

Diverse sampling systems have been developed with differences in parameters like sample volume and the fraction of exhaled air collected. In adults, each breath typically consists of around 500 mL of air. The initial 150 mL corresponds to the dead space, comprising air from the mouth and the surrounding environment, which is not involved in blood gas exchange. The subsequent 350 mL constitutes the alveolar portion, where the air has come into contact with the blood in the lungs. Capturing the alveolar breath, obtained at the end of exhalation, is more advantageous as it avoids dilution by dead space air (White and Fowler, 2018).

##### 3.2.2.1 Sampling bags

Sampling bags are widely used to collect exhaled air for studying VOCs. Patients are asked to fast for at least 6 h and rest for 10 mins before breathing normally through a filter to purify inhaled air or medical air. Sometimes, patients wear a nose clip to ensure that only oral cavity air is collected. Consistency in respiratory parameters, such as the breathing route, is crucial to avoid bias in the clinical interpretation of exhaled VOCs patterns (Sukul et al., 2017).

Subjects are often asked to hold their breath for 10–30 s and then exhale deeply to fill the sampling bag (Koureas et al., 2020). Different types of bags, including Tedlar^®^ (made of polyvinyl fluoride), Flexfilm^®^ (unknown polymer), and Kynar^®^ (made of polyvinylidene difluoride) have been compared (Mochalski et al., 2013a). A study by Mochalski *et al.* (Mochalski et al., 2013a) found that Tedlar^®^ bags provide the least background emission, while Flexfilm^®^ bags provide the highest. The authors recommend storing breath samples for up to 6 h at a cold temperature (4°C), in pre-conditioned Tedlar^®^ bags (flushed with an inert gas).

Cleaning the sampling bags with pure nitrogen, (Caldeira et al., 2012; Mochalski et al., 2013a; Amal et al., 2016; Gashimova et al., 2019; Koureas et al., 2020), helium (Murtada et al., 2021) or humidified zero-air (Be et al., 2008). the main drawback of sampling bags is their storage time, with Mochalski *et al.* (Mochalski et al., 2013a) recommending storing the bags at 4°C and to not extract VOCs from the bags beyond 6 h after sampling.

##### 3.2.2.2 Commercial devices

The Breath Collection Apparatus (BCA, Menssana Research) is a tube-based device where patients blow for 2 min, wearing a nose clip to collect only oral cavity air. Volatile compounds are trapped on activated carbon sorbents inside the tube (Phillips et al., 2019). To our knowledge, published VOCs limits of detection and GC-MS analysis for this system are lacking in the literature.

The Bio-VOC Sampler^®^ (Markes) is designed to collect end-tidal breath, mainly composed of endogenous compounds. The patient blows through a disposable mouthpiece into an inert plastic container with a volume of 175 mL. It samples 88 mL of alveolar air, making it suitable for detecting high-concentration VOCs in the ppm range (Kwak et al., 2014). It has been used for the determining of nitric oxide levels in exhaled air (Henderson and Matthews, 2002). Yet, it is not recommended for identifying unknown biomarkers in low concentrations (<ppb) in breath samples.

The ReCIVA^®^ facemask by Olwstone Medical is an end-tidal breath collection device with an infrared CO_2_ sensor and Tenax^®^ adsorbent cartridges. This commercial device has been well thought out for medical research applications (De Vi et al., 2020; Holden et al., 2020). It allows reproducible and precise breath sampling with reduced potential loss of compounds. However, it is an expensive device, and the same facemask cannot be used for multiple patients. One study using a very small cohort (three patients) suggests that the device may be less sensitive than Tedlar^®^ bags (Gilio et al., 2020).

In summary, each commercial device has its advantages and limitations in breath sampling, making them suitable for specific research applications.

#### 3.2.3 Extraction methods for GC-MS analysis

In this section, the main techniques for preparing gaseous samples for VOCs analysis by GC-MS are presented.

##### 3.2.3.1 Solid phase Micro extraction (SPME)

SPME is a well-established technique developed in 1990 by Pawliszyn *et al.* (Catherine and Janusz, 1990). It is widely used for VOCs analysis. This extraction technique relies on specific polymers like PDMS, Carboxen, and Polyacrylate, which are incorporated into a 1–2 cm long fiber (Reyes-Garcés et al., 2018) with an adsorbent phase ranging from 7 to 100 µm.

SPME protocols involve exposing the fiber to the sample either through immersion for liquid samples or in the headspace of the sample container. After extraction, the VOCs are thermally desorbed from the fiber in the GC-MS inlet for analysis.

A more recent advancement is Thin-Film Solid Phase Microextraction (TF-SPME), developed in 2003 by Bruheim *et al.* (Bruheim et al., 2003). This technique operates on the same principle as classical SPME but utilizes a thin-film surface, about 200 mm^2^ in size, which is approximately 20 times that of a conventional 100 µm SPME fiber.

Murtada *et al.* (Murtada et al., 2021), combined TF-SPME with Tedlar^®^ bags for collecting volatile compounds from exhaled air. They introduced a Carboxen/PDMS film into a 1 L Tedlar bag, which was cleaned with helium to eliminate contamination prior to the sample collection. The filled bag was kept for 3 h at 25°C. Subsequently, the fiber was removed and analyzed by thermal desorption and GC-MS.

##### 3.2.3.2 Trapping in the adsorbent phase

Trapping in sorbent phases is a commonly used method for samples collected in sampling bags. A recent review by Westphal et al., 2023 (Westphal et al., 2022) offers valuable guidelines on using sampling bags and adsorbent phase for thermal desorption and GC-MS analysis. These guidelines encompass essential instrumental parameters, ensuring effective and efficient analysis.

##### 3.2.3.3 Needle Trap Device

Needle Trap Device (NTD) is an extraction trap consisting of a sorbent material packed inside a needle, used as an extraction trap (Pizzini et al., 2018; Monedeiro et al., 2021). This principle closely resembles trapping in sorbent phases. VOCs from the sampling bag or device are transferred to the NTD using a sampling pump. Once extracted, the needle is inserted into a thermal desorption unit, such as a thermal desorption tube, for further analysis.

##### 3.2.3.4 Dynamic headspace

Dynamic Headspace (DHS) is a dynamic extraction technique employed for analyzing volatile compounds in a liquid or a solid sample. In this method, the volatile compounds are thermally extracted and trapped in a sorbent phase. The key advantage of DHS is the continuous renewal of analytes to the sorbent phase through an inert gas flow during the extraction process. This ensures that the equilibrium of volatile compounds between the sample and the gas phase (headspace) is never reached.

DHS can be automated or remote and is suitable for various sample types, particularly solid samples with minimal moisture content. For instance, gauze or a Getxent^®^ tubes are suitable sorbent materials for DHS. Notably, DHS was utilized in a volatolomic study to analyze the sebum of patients with Parkinson’s disease (Sinclair et al., 2021). Sterile cotton medical gauze was used to collect sebum from patients’ upper back. The collected volatile compounds on the gauzes were then extracted by DHS at 80°C for 10 min under nitrogen flow and trapped on a Tenax TA adsorbent cartridge maintained at 40°C. Subsequently, the thermal desorption of the Tenax trap and GC-MS analysis differentiated samples from patients and healthy volunteers.

In a recent systematic review by Mitra et al., 2022 (Mitra et al., 2022), 29 papers were selected, covering diverse sampling devices and extraction techniques for body odor analysis. The number of VOCs detected by GC-MS varied also on the study design, the number of participants, and the analytical instrument used. The sensitivity of the different materials and techniques is challenging to evaluate due to the limited availability of quantification of the VOCs in sweat samples. However, studies providing concentration values demonstrated that 2D-GC with SPME (Caldeira et al., 2012) or Trapping in TD tubes (Berna et al., 2021) allow VOCs detection at very low concentrations (pg/L).

In comparing similar sampling protocols, the sensitivity of the analysis appears to be more influenced by the analytical instrument than the extraction method used.

## 4 Discussion

**FIGURE 3:**
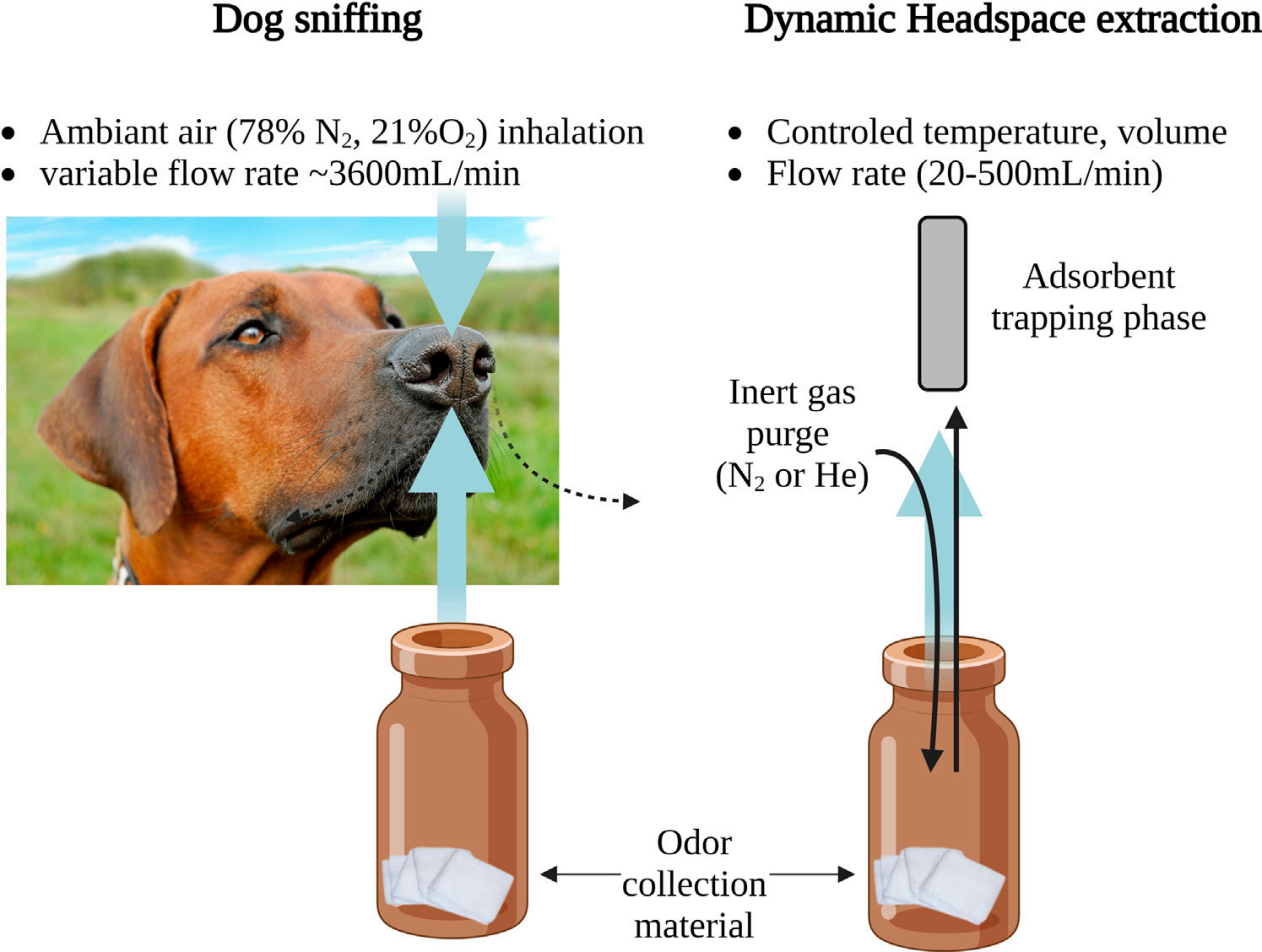
Comparison of dog sniffing and dynamic headspace for GC-MS analysis.

Compared to classical extraction methods discussed in this paper (Section 3.2.3), the canine nose is a natural highly efficient extraction system. The airflow dynamics around a dogs’ nostrils are such that the air is inhaled from the front and exhaled to the side resulting in an impressive volume of approximately 30 mL/s/nostril or an approximate airflow of 3,600 mL/min (Kenneth et al., 2017; Kokocińska-Kusiak et al., 2021). This flow rate is over 7 times higher than the typical values used for human exhaled air extraction from sampling bags or gas purge flow used in dynamic headspace to extract sweat-VOCs from materials (ranging from 20 to 500 mL/min) before thermal desorption GC-MS analysis. To better replicate the accuracy of a dog’s sniffing process, it is recommended to employ dynamic headspace techniques for VOCs collection instead of using static headspace methods. For instance, using DHS to simulate a 15 s dog sniffing in one nostril, a sample volume of 450 mL has to be extracted at a flow rate of 30 mL/s. Figure 3 illustrates the similarities between dog sniffing and dynamic headspace techniques used in GC-MS analysis.

### 4.1 Use of the dog as an analytical tool

Canine olfaction as a diagnostic tool offers many advantages. Firstly, it provides a non-invasive, painless, and potentially inexpensive procedure. The use of canine olfaction as a diagnostic tool has shown promise in detecting specific VOCs patterns in sweat or exhaled air associated with infectious diseases like COVID-19, or cancers. Since domestic dogs are familiar with human odors, they need to be trained to differentiate between body odors from multiple healthy subjects and those combined with disease-specific VOCs. The training process for detection dogs typically involves two steps: generalization, where they are trained to detect specific stimuli, and discrimination: where they learn to differentiate between target odors and distracting samples.

Once properly trained, sniffing dogs, selected based on breed and aptitudes, exhibit remarkable abilities in detecting VOCs at extremely low concentrations, often in the parts per trillion range. Interestingly, the sensitivity of the dog’s olfactive system appears to be on par with, if not greater than, the analytical instrument. For instance, when comparing Limit of Detection (LOD) values, butanethiol is estimated to have an LOD of 0.0003 ppb for canine olfaction (Wackermannová et al., 2016) *versus* 0.03 ppb for GC- MS (Muir et al., 2005). On the other hand, certain VOC like Pyrazine are detected at lower LOD by GC-MS (0.4 ppb) (Mochalski et al., 2013b) compared to dog sniffing, which achieves an LOD of 28 ppb (Wackermannová et al., 2016). However, canine olfaction cannot be compared to analytical instruments such as GC-MS systems since these two detection tools are very different. Indeed, canine olfaction is believed to depend on broadly selective receptors and combinatorial signal processing. One crucial factor to consider is that dogs are living beings and exhibit variability in their responses. Interpretation of canine olfaction analyzes must account for the variability among individual animals and among dog’s breeds (Bauër et al., 2022b).

Moreover, the complexity of the training exercise can increase this variability. Properly training detection dogs to recognize these disease-specific odorants remain a significant challenge. It is essential to ensure that the dogs do not confuse these odors with unrelated stimuli, such as contaminants, hospital odors, or even the sampling device or storage conditions.

Therefore, attention must be given to the experimental study’s design and the collection and storage of training and test samples. Unfortunately, there is a lack of standardization of canine olfaction training, which is evident in many studies. To address this, a well-defined training protocol should be developed, drawing from experiences acquired in forensic sciences (National Institute of Standards and Technology, 2009).

In a medical context, additional considerations come into play. A study conducted by Elliker *et al.* (Elliker et al., 2014) offers valuable recommendations for experimental studies related to cancer diagnosis using canine olfaction. One of the challenges is that detection dogs can identify samples used in previous training sessions based on individuals’ odors or contaminants’ odors. To mitigate this issue, it is recommended not to reuse several times training samples from the same individuals but instead to use pooled samples from different donors to create a variety of odor profiles (unfamiliar samples) (Elliker et al., 2014).

The control of VOCs released from the odor capture devices during dog training is also a critical aspect to consider. Forensic experts have developed a standardized procedure called Controlled Odor Mimic Permeation Systems (COMPS) to address this (Kenneth et al., 2008; Kenneth et al., 2017). The diffusion time of a compound within an odor collection material depends on its chemical properties such as vapor pressure and structure, as well as its affinity with the sorbent material. This process involves two distinct physical mechanisms: adsorption, where molecules adhere to the surface of a solid surface, and absorption, where molecules permeate into a porous surface.

Quantifying the amount of VOCs released from odor collection materials under controlled temperature and humidity conditions is essential to determine their suitable usage time based on the nature of the VOCs being studied. For instance, in a study by Simon *et al.* (Simon et al., 2019), headspace concentrations of 12 VOCs were determined using SPME GC-MS. Analytical standards were doped onto cotton gauze (Dukal brand) and placed onto open containers used for dog training. After 1 hour (the estimated time of a canine training session), the diffusion of VOCs from gauze to the surrounding air was measured. SPME fibers (DVB/CAR/PDMS) were placed 5 cm above the gauze, were exposed for 15 s to the odor source, and subsequently analyzed by TD GC-MS. This process was repeated daily for 9 days. The concentrations of VOCs did not significantly decrease for 7 h, allowing the gauzes to be used for up to 7 training sessions (Simon et al., 2019).

### 4.2 Use of GC-MS as a complementary tool to identify specific-diseases induced VOCs

The analysis of human odor samples by GC-MS applied using untargeted methods allows for the identification of a broad range of volatile molecules. In a first place, to improve the sensibility and the specificity of the analysis, the most relevant materials for sampling and extracting VOCs must be chosen. Commonly used materials like gauze, clothes, and cotton balls cannot be directly used with GC-MS systems and require a preliminary extraction step. The choice of the extraction protocol among various existing headspace techniques does not significantly impact the analysis’s sensitivity. However, it does influence the analysis’s selectivity. For example, bipolar sorbents like Tenax TA or Carboxen are capable of trapping both hydrophile and lipophile VOCs, while PDMS favors the extraction of lipophile compounds. To achieve untargeted analyses, essential for detecting and identifying unknown disease biomarkers, it is recommended to use multiple sorbent phases with different properties. This approach allows for the trapping of a wide range of analytes, enhancing the chances of detecting and characterizing diverse compounds during the analysis. Using PDMS-based devices or Sorbstar^®^ tubes for body odors eliminated the need for an extraction step. These materials can be directly used with a GC-MS system through thermal desorption. By applying optimal thermal desorption parameters, a significant portion of the collected analytes on the sorbent surface can be injected for analysis.

After optimizing the parameters for VOCs sampling and extraction, the later step is the analysis. In the field of metabolomics, untargeted GC-MS methods are prevalent. Then the data analysis can be carried out using various methods. The criteria for selecting peak (quality of peak height or range of m/z value), the variables values (peak area, peak height, intensity value of MS or abundance value of TIC) and the choice of data preprocessing method, are key parameters in the data treatment (Md Ghazi et al., 2022). Data preprocessing includes peak detection, baseline correction, chromatographic alignment, deconvolution, feature filtration, missing value replacement, normalization, ratio selection and classification. Typically, when applying metabolomics to disease diagnosis, the primary objective is to predict specific disease classes through the use of multivariate methods (Feizi et al., 2021).

Principal Component Analysis (PCA) is commonly applied in a first place to provide an overview of the data, detect outliers, groups, and patterns. Subsequently, classification can be performed using linear methods such as Partial Least Squares (PLS) and Orthogonal Partial Least-Squares (OPLS), or non-linear methods like Support Vector Machine (SVM) (Feizi et al., 2021). Linear methods are preferred for the analysis of a small sample size relative to a high number of variables, whereas non-linear methods are dedicated to large datasets. For instance, Caldeira *et al.* (Caldeira et al., 2012), applied of GC×GC–ToFMS (Comprehensive Two-Dimensional Gas Chromatography coupled with Time-of-Flight Mass Spectrometry) to the analysis of exhaled air samples from 32 children with allergic asthma (of which 10 also presented with allergic rhinitis) and 27 control children. Subsequently, PLS-Discriminant Analysis in conjunction with Cross Validation, was conducted to identify compounds that might be associated with oxidative stress, inflammatory processes, or other cellular phenomena characteristic of asthma. Following this process, a robust model with a classification rate of 96% is obtained. Within the asthmatic population, a distinctive profile of six VOCs was identified: nonane, 2,2,4,6,6-pentamethylheptane, decane, 3,6-dimethyldecane, dodecane, and tetradecane.

In order to explore potential future clinical applications, targeted methods have also to be developed to reduce the data processing time and enhances the method’s suitability for diagnosis purposes. Monedeiro *et al.* (Monedeiro et al., 2021) went in that direction by quantifying 29 target VOCs that have been previously reported as potential biomarkers of lung diseases in breath (lung cancer, chronic obstructive pulmonary disease, and asthma). Multinomial logistic regression (MLR) has been utilized to assess the relationship between the concentrations of the nine most discriminative targets (2-propanol, 3-methylpentane, (E)-ocimene, limonene, m-cymene, benzonitrile, undecane, terpineol, and phenol) as input variables. This analysis yielded an average overall accuracy of 95.5% for the prediction of multiple classes.

As discussed in Section 3.1, canine olfaction can be employed for simple binary classification of samples. Indeed, it would be intriguing to use GC-MS as an assessment tool for the dog. However, in the current state of our knowledge, we cannot assure what stimuli the dogs are actually responding to. The biomarkers identified by GC-MS might not be the same as those detected by the dogs. Furthermore, it is possible that the LODs of even the most advanced analytical instruments may not allow for the detection of molecules perceived by the dogs.

Nevertheless, studies in the field of analytical chemistry on the olfactory characteristics of pathologies serves as a valuable complement and may contribute to a more comprehensive understanding and standardization of experiments involving detection dogs (Bauër et al., 2022b).

### 4.3 Design of an ideal device compatible with both canine olfaction and GC-MS analysis

**FIGURE 4:**
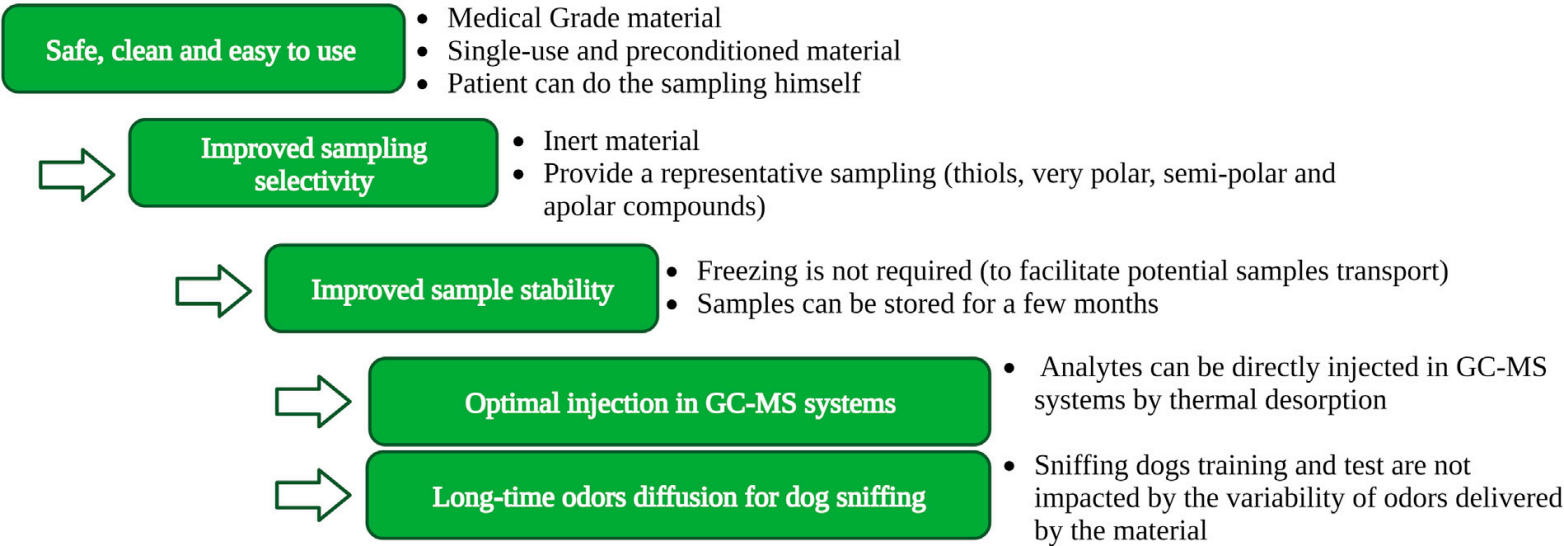
A conceptual design for an ideal sampling device enabling for simultaneous analysis of body odors by canine olfaction and GC-MS.

The design of an innovative, ideal device should consider several key elements, as resumed in Figure 4. These elements should be considered as recommendations for future research in the field of medical odor analysis.

1. Practical aspects: To ensure consistency and minimize variability in experimental studies involving numerous subjects, the device should have a user-friendly sampling protocol. An ideal device would come pre-conditioned to be free of contaminants and single-use to avoid cross-contamination. It should be packed in an inert vacuum-sealed individual bag before and after sampling.
2. Sampling selectivity: To improve dogs’ performances, the sampling device must be capable of collecting volatile compounds from diverse chemical families with different physicochemical properties. Balancing the ability to trap both apolar and polar compounds is essential, as valuable information could be lost if the collection device favors only one type of compound. Preserving highly reactive compounds like thiol molecules before analysis would be a significant advancement in medical diagnosis applications involving body odors (Schroeder, 2015).
3. Sample stability: Care must be taken when using absorbing materials like cellulose fibers (gauze, surgical facemasks) as they can absorb VOCs, humidity, and non-volatile compounds. Cold storage prior to analysis is necessary to prevent molecule degradation. In contrast, sorbent materials like adsorbent polymers are inert and do not require freezing but should be stored in a cold place to avoid the desorption of volatile compounds prior to analysis. The ideal device should allow long-time storage (several months) to accommodate experimental studies involving patients recruited and sampled over an extended period. Analyzing all samples in the same batch at the end of the study can help reduce instrumental variability.
4. Compatibility with thermal desorption: An ideal material would be compatible with direct thermal desorption prior to GC-MS analysis to enhance the analysis’s sensitivity by minimizing VOCs loss from extraction steps.
5. Reproducibility of odor release: Consistency in the amount of odor released from the sampling material throughout the training session or the test time is essential for accurate canine olfaction and GC-MS analysis.

## 5 Conclusion and perspectives

This bibliographic study explores the tools to comprehend odor capture techniques for both canine olfaction and GC-MS systems. The analysis of odors in a medical context is a relatively recent and diverse field, with numerous proofs of concept utilizing detection dogs or GC-MS. Interestingly, none of the studies reviewed have been replicated in other laboratories, and their results vary significantly. Notably, the only study on Parkinson’s disease was repeated with an increased patient cohort and yielded contradictory results compared to the initial study, serving as a crucial proof of concept odor (Trivedi et al., 2019a; Sinclair et al., 2021).

A key observation is the lack of standardization in sample collection protocols for both dogs and GC-MS studies. Training sniffing dogs necessitates an odor capture device, which can take the form of a sampling bag for patients to blow into, a gauze for sweat collection, or a passive/active polymer-based device to adsorb human VOCs. However, questions arise concerning the preservation and potential reuse of these odor-capturing devices, under specific conditions. Although this review does not offer a definitive answer to this question, it does provide valuable guidelines for consideration in future studies.

To potentially identify volatile biomarkers of pathologies detected by dogs, conducting GC-MS analysis alongside canine olfaction, using replicate samples and direct thermal desorption or multi-sorbent extraction may prove instrumental. This approach could eventually pave the way for significant advancements in the field of medical odor analysis.

Perhaps one of the most conceivable field applications utilizing sniffing dogs, would be the detection of infectious diseases during a pandemic. Indeed, in an observational study related to SARS-CoV-2, Guest *et al.* (Guest et al., 2022), reported that 2 dogs could screen 300 people in 30 min. This hypothesis makes it possible to imagine using canine olfaction in airports before embarking in a plane. In this scenario, only individuals marked as positive by the dogs would be tested afterwards by PCR tests.

In isolated settings, where access to gold standards diagnosis instruments is difficult, bringing detection dogs appears to be a promising solution for early and rapid detection of pathologies. In our knowledge, there are no established applications using detection dogs in this context. The limitations of this approach are well outlined in the review by Bauer *et al.* (Bauër et al., 2022b). These drawbacks notably include the associated high costs of employing qualified personnel for such applications, and the challenge of consistently sourcing samples for dogs’ trainings, to maintain their performances over time (Bauër et al., 2022b).
