# Supplementary material for: Unraveling the potential of breath and sweat VOC capture devices for human disease detection: a systematic-like review of canine olfaction and GC-MS analysis
Source: Front Chem. 2023 Nov 1;11:1282450. doi: 10.3389/fchem.2023.1282450 (PMC10646374; doi:10.3389/fchem.2023.1282450)
Supplement: Supplementary file 1 [file Table1.DOCX]

**Table 1 : Sweat analysis by GC-MS**

| **body area** | **Sampling device** | **Sampling method** | **Extraction method** | **Adsorbent phase** | **Desorption method** |  | **Sample storage conditions** | **Analysis method** | **GC-MS system** | **GC-column** | **Sensibility** | **Ref.** |
| --- | --- | --- | --- | --- | --- | --- | --- | --- | --- | --- | --- | --- |
| wrist and ankle | Custom sampler made of a 25cm length tube made of medical grade PDMS (0.64 mm OD × 0.3 mm ID, Sil-Tec®). | passive sampling for 1h in direct contact with the skin. Each subject wear 2 tubes as anklets and 2 tubes as bracelets. Mylar® reflective sheeting (Hydroponic), 35 cm × 3 cm, is used to cover the samplers | n/a | n/a | **Thermal desorption** at 250°C in splitless mode for 30s (PDMS sampler is inserted into a glass inlet liner (Agilent)) |  | stored in aluminum foil at 4°C for no more than 48h | GCxGC-TOFMS | LECO Pegasus® 4D GC×GC-TOFMS system | 1D column : Rxi-5Sil (Restek) 30m × 0.25mm × 0.25μm  2D column : Rxi-17Sil (Restek) 1m × 0.25mm × 0.25µm | **not specified** | [1] |
| forehead | PDMS patch (5mm x 15mm x 0,45mm) sandwiched between two layers of thin, flexible stainless-steel mesh | PDMS patch is held in place by a conditioned cotton-wool pad on the subject’s forehead for 30min | n/a | n/a | **Thermal desorption** at 180°C for 10min. **Cryo-focusing** at -10°C. Injection splitless at 300°C for 5min |  | PDMS patches are stored into empty inert coated stainless steel thermal desorption tubes at -80°C for no longer than 21 days | GC-MS | HP5890 GC coupled to a Fisons Trio 1000 quadrupole mass spectrometer | DB-5 MS (Agilent) 60m x 0.25mm x 0.25µm | **170pg/cm to 200pg/cm** | [2] |
| forearms and abdomen | PDMS patches (20mm × 15mm × 0.45mm) | sampling in direct contact with the skin; patch are placed on the skin and covered with a cotton wool pads, fixed with microporous tape for 5min to 120min | n/a | n/a | **Thermal desorption** at 180°C for 5min. **Cryo-focusing** at -10°C. Injection splitless at 300°C for 3min |  | stored into unpacked Silcosteel^®^ treated thermal desorption tubes (C-TBE10; Markes) sealed with stainless steel caps (SS-400-C; Swagelok), at 4°C in airtight glass containers **no longer than 24h** | GC-MS | GC (not specified)-ion trap MS (Varian, 2200) | DB-5MS (Agilent) 60m x 0,25mm x 0,25µm | **50pg to 100ng per sample (relative estimation)** | [3] |
| upper back, forearm and back thigh | 6, 11 and 17mm diameter PDMS round patches with a thickness of 254 µm, sandwiched between two layers of thin, flexible stainless steel mesh | headspace passive sampling for 60min | n/a | n/a | **Thermal desorption** at 250°C for 3min. **Cryo-focusing** at -120°C. Injection splitless at 280°C |  | stored no **longer than 72h** | GC-MS | GC 6890 (Agilent) coupled to a 5973 quadrupole mass spectrometer (MS) (Agilent) | RK13870 (Restek) 30m x 0,32mm x 1,8µm | **not specified** | [4] |
| arms | Twister (PDMS)-coated stir bars (10 mm,0.5 mm in film thickness, 24 ml PDMS volume; Gerstel GmbH) | active sampling in direct contact with the skin | n/a | n/a | **Thermal desorption** at 280°C for 10min. **Cryo-focusing** at −60°C. Injection at 280°C for 10min |  | stored at 4°C for 14 days | GC-MS | Agilent 6890N GC connected to 5973i MSD MS | DB-5MS (Agilent) 20m x 0,18mm x 0,18µm | **not specified** | [5] |
| axilla | Twister (PDMS)-coated stir bars (10 mm,0.5 mm in film thickness, 24µL PDMS volume; Gerstel GmbH) | active sampling in direct contact with the skin | n/a | n/a | **Thermal desorption** at 250°C for 3min. **Cryo-focusing** at −80°C. Injection at 280°C for 10min |  | stored at 4°C for 20 days | GC-MS | Agilent 6890N GC connected to 5973i MSD MS | DB-5MS (Agilent) 20m x 0,18mm x 0,18µm | **not specified** | [6] |
| inner arm and chest | 132 mg of 20/35 meshed poly(2,6-diphenylphenylene oxide) | sampling for 1h in direct contact with the skin | n/a | n/a | **Thermal desorption** at 300°C for 15min |  | stored in vials closed and wrapped with Parafilm at 4°C, up for 8 months | GC-MS | Shimadzu QP–2010 | SLB-5ms (Sigma-Aldrich) 30m x 0,25mm x 0,5mm | **ppb** | [7] |
| hand | DUKAL brand, sterile, 2 x 2, 8ply, gauzes sponges | active sampling in direct contact with the skin (palm hands) | SPME at room temperature for 21h | CAR/DVB/PDMS | **Thermal Desorption** at 250°C |  | stored at room temperature for 24 h | GC-MS | Agilent 6970 GC with a 5973 MS | HP5-MS (Agilent) 30 m x 0.25 µm x 0.25 mm | **not specified** | [8] |
| feet | a strip of cotton wool (3.0 g) | sampling in direct contact with the feet skin (cotton placed in the socks) for 6h | SPME 10 min at 55°C | polyacrylate (85 mm) 100-mm-long fiber | **Thermal desorption** at 230°C for 3min |  | not specified | GC-MS | GC Star 3400 CX (Varian) coupled with an ion-trap mass spectrometer detector Saturn 2000 (Varian) | ZB-FFAP (Phenomenex) 30m x 0,25m x 0,25µm | **2-10nmol for fatty acids** | [9] |
| genitourinary area | gauze made of cotton and cellulose, of 20x13 cm dimension | sampling in direct skin (genito-urinary area) overnight | Headspace 100°C, 70 rpm for 30 min | n/a | Injection splitless 260°C |  | not specified | GC-MS | G890 N Network GC coupled to a 5973 Network MSD (Agilent) | DB-1701 (Agilent) 60m x 250 mm x 0.25mm | **not specified** | [10] |
| palm of hands | gauze | sampling in direct contact with the skin (2cm square area of the palm is wiped for 1 min with 0.1 g of dry gauze) | SPME at 50°C for 45min | PDMS/DVB | **Thermal desorption** at 230°C |  | stored 24h at 4°C | GC-MS | Shimadzu QP–2010 | DB-1 (J&W) 60m × 0.25mm x 1.0µm | **LOD for 2-nonenal: 2.4pg/cm^2^/h** | [11] |
| upper back | medical gauze | active sampling by swabbing the gauze on the skin to collect the sebum and the sweat (sampling time not specified) | DHS; incubation for 5 min at 60 °C, trapping by purging 500 mL of the sample headspace at 50 mL/min with dry nitrogen through an adsorbent tube kept at 40°C | Tenax TA | **Thermal desorption** at 250°C for 5min, split ratio 1:10; **Cryo focusing** at 10°C, injection at 250°C for 10min |  | stored at -80°C in inert plastic bags | GC-MS | GC 7890B coupled to a MSD 5977B (Agilent) | HP-5MS Ultra inert (Agilent) 30m × 0.25mm × 0.25μm | **not specified** | [12] |
| armpit | absorbent pads | passive sampling (pads are attached via stainless steel poppets in pre-cleaned T-shirts) | HSSE in 250mL Scott-Duran GLS80 bottles with a PDMS stir bar (1cm long, 1mm thickness) at 60°C for 2h | PDMS | **Thermal desorption** at 250°C for 5min, splitless; **Cryo focusing** at -120°C, injection at 250°C for 5min with a split ratio 1:5 |  | stored in bags and vacuum-sealed at -28°C | GCxGC-TOFMS | Pegasus® 4D GCxGC-TOFMS (Leco) | 1D column: Rxi-5MS (Restek) 30m x 0,25mm x 0,5µm; 2D column : RTX-200 (Restek) 2m x 0,15mm x 0,15µm | **not specified** | [13] |
| hand | Sterile cotton gauze pads (100% cotton) Dukal |  | SPME; equilibration at 50°C for 24h, extraction for 15h | 2cm fiber, 50/30µm DVB/CAR/PDMS | **Thermal desorption** at 270°C for 5min in splitless mode |  | stored in the cleaned 10 mL vials, sealed and secured with parafilm around the screw cap opening | GC-MS | GC 8890 coupled to a 5977B MSD (Agilent) | HP5-MS UI (Agilent) 15m x 0,25mm x 0,25µm | **not specified** | [14] |
| palm of hands | Gauze pads were DUKAL brand, 100% cotton, sterile, 2 × 2, 8ply, gauze sponges | Subjects hold the gauze between the palms of their hands as they walked outdoors for 10 min | SPME at room temperature for 21h | 50/30µm DVB/CAR/PDMS | **Thermal Desorption** at 250°C |  | stored in sealed 10mL glass vial for 24h prior to extraction at ambient temperature | GC-MS | GC 6970 (Agilent) with a 5973-mass selective detector (MS) | HP-5MS (Agilent) 30m x 0,25mm x 0,25µm | **not specified** | [15] |
| armpit | Dukal brand, sterile, 2x2, 8-ply, gauze sponges | Subjects wiped a gauze on their armpit after 30min of outdoors physical exercised | SPME at room temperature for 15h | 50/30µm DVB/CAR/PDMS | **Thermal Desorption** at 250°C |  | stored in sealed 10mL glass vial for 24h prior to extraction at ambient temperature | GC-MS | GC 6970 (Agilent) with a 5973-mass selective detector (MS) | HP-5MS (Agilent) 30m x 0,25mm x 0,25µm | **not specified** | [16] |
| bust | cotton-shirts | passive sampling; subjects wear the shirt for 3 days; a rectangular piece 20 x 30cm is cut and stored in a 10L Tedlar® bag | DHS using a sampling pump; The flow rate of the pump was maintained at 1.8L/min while air deodorized with activated carbon was supplied to the bag to keep the capacity at 10L.Headspace collection was performed at 23°C for 18 h | TENAX-TA (GL Science) | **Liquid desorption** with 10mL of diethyl ether |  | stored in a 10L Tedlar® bag at room temperature in a dark place for no longer than one day | GC-MS | G1800A GCD system (Hewlett-Packard) | INNOWAX (Hewlett-Packard) 60m x 0,25mm x 0,25µm | **ppm** (concentrations in ng per mg skin surface lipids) | [17] |

[1] M. Wooding, E. R. Rohwer, and Y. Naudé, “Chemical profiling of the human skin surface for malaria vector control via a non-invasive sorptive sampler with GC×GC-TOFMS,” *Anal Bioanal Chem*, vol. 412, no. 23, pp. 5759–5777, Sep. 2020, doi: 10.1007/s00216-020-02799-y.

[2] H. J. Martin, M. A. Turner, S. Bandelow, L. Edwards, S. Riazanskaia, and C. L. P. Thomas, “Volatile organic compound markers of psychological stress in skin: A pilot study,” *J Breath Res*, vol. 10, no. 4, Nov. 2016, doi: 10.1088/1752-7155/10/4/046012.

[3] S. Riazanskaia, G. Blackburn, M. Harker, D. Taylor, and C. L. P. Thomas, “The analytical utility of thermally desorbed polydimethylsilicone membranes for in-vivo sampling of volatile organic compounds in and on human skin,” *Analyst*, vol. 133, no. 8, pp. 1020–1027, 2008, doi: 10.1039/b802515k.

[4] R. Jiang, E. Cudjoe, B. Bojko, T. Abaffy, and J. Pawliszyn, “A non-invasive method for in vivo skin volatile compounds sampling,” *Anal Chim Acta*, vol. 804, pp. 111–119, Dec. 2013, doi: 10.1016/j.aca.2013.09.056.

[5] D. J. Penn *et al.*, “Individual and gender fingerprints in human body odour,” *J R Soc Interface*, vol. 4, no. 13, pp. 331–340, Apr. 2007, doi: 10.1098/rsif.2006.0182.

[6] Y. Xu *et al.*, “Comparison of human axillary odour profiles obtained by gas chromatography/mass spectrometry and skin microbial profiles obtained by denaturing gradient gel electrophoresis using multivariate pattern recognition,” *Metabolomics*, vol. 3, no. 4, pp. 427–437, Dec. 2007, doi: 10.1007/s11306-007-0054-6.

[7] R. Vishinkin *et al.*, “Profiles of Volatile Biomarkers Detect Tuberculosis from Skin,” *Advanced Science*, vol. 8, no. 15, Aug. 2021, doi: 10.1002/advs.202100235.

[8] A. M. Curran, P. A. Prada, and K. G. Furton, “The differentiation of the volatile organic signatures of individuals through SPME-GC/ms of characteristic human scent compounds,” *J Forensic Sci*, vol. 55, no. 1, pp. 50–57, Jan. 2010, doi: 10.1111/j.1556-4029.2009.01236.x.

[9] A. Caroprese, S. Gabbanini, C. Beltramini, E. Lucchi, and L. Valgimigli, “HS-SPME-GC-MS analysis of body odor to test the efficacy of foot deodorant formulations,” *Skin Research and Technology*, vol. 15, no. 4, pp. 503–510, Nov. 2009, doi: 10.1111/j.1600-0846.2009.00399.x.

[10] M. Rodríguez-Esquivel *et al.*, “Volatolome of the Female Genitourinary Area: Toward the Metabolome of Cervical Cancer,” *Arch Med Res*, vol. 49, no. 1, pp. 27–35, Jan. 2018, doi: 10.1016/j.arcmed.2018.04.004.

[11] K. Saito, Y. Tokorodani, C. Sakamoto, and H. Kataoka, “Headspace solid-phase microextraction/gas chromatography–mass spectrometry for the determination of 2-nonenal and its application to body odor analysis,” *Molecules*, vol. 26, no. 19, Oct. 2021, doi: 10.3390/molecules26195739.

[12] D. K. Trivedi *et al.*, “Discovery of Volatile Biomarkers of Parkinson’s Disease from Sebum,” *ACS Cent Sci*, vol. 5, no. 4, pp. 599–606, Apr. 2019, doi: 10.1021/acscentsci.8b00879.

[13] M. A. M. Smeets *et al.*, “Chemical fingerprints of emotional body odor,” *Metabolites*, vol. 10, no. 3, Mar. 2020, doi: 10.3390/metabo10030084.

[14] J. Crespo-Cajigas *et al.*, “Investigating the Use of SARS-CoV-2 (COVID-19) Odor Expression as a Non-Invasive Diagnostic Tool—Pilot Study,” *Diagnostics*, vol. 13, no. 4, p. 707, Feb. 2023, doi: 10.3390/diagnostics13040707.

[15] A. M. Curran, C. F. Ramirez, A. A. Schoon, and K. G. Furton, “The frequency of occurrence and discriminatory power of compounds found in human scent across a population determined by SPME-GC/MS,” *J Chromatogr B Analyt Technol Biomed Life Sci*, vol. 846, no. 1–2, pp. 86–97, Feb. 2007, doi: 10.1016/j.jchromb.2006.08.039.

[16] A. M. Curran, S. I. Rabin, P. A. Prada, and K. G. Furton, “Comparison of the volatile organic compounds present in human odor using SPME-GC/MS,” *J Chem Ecol*, vol. 31, no. 7, pp. 1607–1619, Jul. 2005, doi: 10.1007/s10886-005-5801-4.

[17] S. Haze *et al.*, “2-Nonenal Newly Found in Human Body Odor Tends to Increase with Aging.”
