# Supplementary material for: Unraveling the potential of breath and sweat VOC capture devices for human disease detection: a systematic-like review of canine olfaction and GC-MS analysis
Source: Front Chem. 2023 Nov 1;11:1282450. doi: 10.3389/fchem.2023.1282450 (PMC10646374; doi:10.3389/fchem.2023.1282450)
Supplement: Supplementary file 2 [file Table2.DOCX]

Table 2: Extraction methods for GC-MS analysis breath collected in sampling bags.

| Extraction method | Adsorbent phase | Protocol | Desorption method | Sample storage conditions | Analysis method | GC-MS system | GC-column | Sensibility | Ref. |
| --- | --- | --- | --- | --- | --- | --- | --- | --- | --- |
| NTD | PDMS, Carbopack-X, Carboxen 1000 | air pump, 30mL/min | **Thermal Desorption** at 260°C (2min) | not specified | GC-MS | 6890 A gas chromatograph coupled with a 5975 Inert XL MSD (Agilent) | DB-624 (Agilent) 60m × 0.32mm × 1.8µm | **ppb** | [1] |
| NTD | 80 mg Carbotrap-B and 260 mg Carbopack-X | air pump, 30mL/min | **Thermal Desorption Unit TD-100** (Markes International). Desorption at 320 °C for 10 min using helium as an inert carrier gas with a flow rate of 50 mL/min. **Cryo-focusing** onto a cold trap kept at −20 °C filled with graphitized carbon blacks. The temperature of the cold trap is then rapidly raised to 340 °C hold for 2 min | samples are extracted immediately after sampling and analyzed on the same day (within a few hours) | GC-ToF-MS | 7890B Gas Chromatograph (Agilent) coupled with a Bench-TOFdx time-of-flight mass spectrometer (Five Technologies) | apolar column Restek-Q-Bond 30m x 0.25mm x 8μm | **ppb** | [2] |
| SPME | PDMS | 60min at 37°C | **Thermal desorption** at 250°C in a splitless mode (3min) | not specified | GC-MS | Gas Chromatograph Mass Spectrometer GCMS-QP2010/ PLUS (Shimadzu) | Rtx-1 (Restek) 30m × 0.25mm× 0.25µm | **not specified** | [3] |
| SPME | Carboxen, PDMS | 10min at 40°C | **Thermal desorption** at 290°C in a splitless mode (1 min) | not specified | GC-MS | 7890 Gas Chromatograph (Agilent) coupled with a mass selective detector MSD type 5975C (Agilent) | PoraBond Q column (Varian) 25m × 0.32mm x 5μm | **ppb** | [4] |
| SPME | DVB, Carboxen, PDMS | 60min at 22°C | **Thermal desorption** at 250°C in a splitless mode (2min) | storage at 22°C for no longer than 6 hours | GCxGC-ToF-MS | GC 7890A (Agilent) with a dual stage jet cryogenic modulator (licensed from Zoex) and a secondary oven coupled to a high-speed ToF mass spectrometer (LECO) | 1D column: HP-5 column (J&W Scientific Inc.) 30m × 0.32mm x 0.25µm 2D column: DB-FFAP (J&W Scientific Inc.) 0.79m × 0.25mm x 0.25µ | **ppq (pg/L)** | [5] |
| SPME | Carboxen, PDMS | 25min at room temperature | **Thermal desorption** at 270°C in a splitless mode (5min) | samples were analyzed within 6h | GC-MS | Finnigan Trace GC Ultra coupled to a Polaris Q Quadrupole Ion Trap GC-MS system | DB-624 (Agilent) 30m × 0.25mm × 1.4µm | **ppt** | [6] |
| TF-SPME | PDMS Thin-Film, 5% Carboxen | 3h at 25°C | **Thermal Desorption** at 270°C for 5 min in tapered glass TD tube (5 mm I.D) at 270°C with a helium flow rate of 60mL/min. **Cryo-focusing** at -120°C in a cooled injection system (CIS). The CIS is then heated to 270°C at 12°C/s | Samples are analyzed immediately after the 3h of extraction | GC-MS | GC-MS 6890/5973(Hewlett Packard) | SLBTM-5MB (Sigma-Aldrich) 30m × 0.25mm × 0.25μm | **ppb** | [7] |
| Trapping in TD tube | Carbotrap C, Carbopack C | air pump | **Thermal Desorption** at 250°C for 8min with helium flowing at 10mL/min. **Cryo-focusing** at -150°C. Injection at 250°C over 1.5min | not specified | GC-MS | HP 5890 Series II Plus GC coupled to 5972 MSD (Hewlett-Packard) | HP5MS 30m x 0.25mm x 0.25µm | **not specified** | [8] |
| Trapping in TD tube | Tenax | air pump, 250mL/min | **Thermal Desorption** at 300°C for 3 minutes. **Cryo-focusing** into a Tenax TA cold trap at −150°C, which was heated after 2 minutes to 280°C at 20°C/s | storage at 4°C for no longer than 2 weeks | GC-MS | 6890 N GC (Agilent) coupled to a quadrupole mass spectrometer 5975 MSD (Agilent) | VF1-MS 30m × 0.25mm x 1 µm | **ppb** | [9] |
| Trapping in TD tube | Tenax TA | air pump, 100mL/min | **Thermal Desorption** at 270°C for 5 min under 60mL/min flow. **Cryo-focusing** on a Tenax cold trap at 0°C; injection in splitless mode at constant 3mL/min total flow | stored at 4 °C until analysis | GC-MS and electronic nose | GCMS-QP2010 (Shimadzu) | SLB-5 MS (Sigma-Aldrich) 30m x 0.25mm x 0.5 | **ppb** | [10] |
| Trapping in TD tube | Tenax, Carbograph, Carboxen | air pump, 200mL/min | **Thermal Desorption** at 270°C for 10min. C**ryo-focusing** at 10°C. The cold trap is subsequently heated to 300°C | storage at 4°C for no longer than 2 weeks | GCxGC BenchTOF-MS | 7890B GC (Agilent) fitted with a flow modulator and a three-way splitter plate coupled to a flame ionization detector and a time-of-flight mass spectrometer with electron ionization (SepSolve). | 1D column: Stabilwax (Restek) 30m × 0.25mm × 0.25μm 2D column: Rtx-200 MS (Restek) 5m × 0.25mm × 0.1μm | **ppq (pg/L)** | [11] |
| Headspace | no adsorbent phase | Gas Syringe 50 mL/min and flow path temperature of 150 ℃ | **Thermal Desorption;** a multiple channel thermal desorption system (UNITYxrTM) with an auto-sampler CIA Advantage-xrTM (Markes) is used to sample 100 mL of exhaled breath from each of the Tedlar bags at a flow rate of 50 mL/min and flow path temperature of 150 ℃ | room temperature no longer than 24h | GC-MS | Trace GC-Ultra gas chromatograph attached to an ISQ Mass Spectrometer (GC-MS, Thermo Scientific) | Rtx-VMS (Restek) 30m × 0.25mm x 1.40μm | **ppm** | [12] |

[1] F. Monedeiro, M. Monedeiro-Milanowski, I. A. Ratiu, B. Brożek, T. Ligor, and B. Buszewski, “Needle trap device-gc-ms for characterization of lung diseases based on breath voc profiles,” *Molecules*, vol. 26, no. 6, Mar. 2021, doi: 10.3390/molecules26061789.

[2] A. Pizzini *et al.*, “Analysis of volatile organic compounds in the breath of patients with stable or acute exacerbation of chronic obstructive pulmonary disease,” *J Breath Res*, vol. 12, no. 3, Mar. 2018, doi: 10.1088/1752-7163/aaa4c5.

[3] Y. Wang *et al.*, “The analysis of volatile organic compounds biomarkers for lung cancer in exhaled breath, tissues and cell lines,” *Cancer Biomarkers*, vol. 11, pp. 129–137, 2012, doi: 10.3233/CBM-2012-0270.

[4] P. Mochalski, J. King, M. Haas, K. Unterkofler, A. Amann, and G. Mayer, “Blood and breath profiles of volatile organic compounds in patients with end-stage renal disease,” *BMC Nephrol*, vol. 15, no. 1, pp. 1–14, Mar. 2014, doi: 10.1186/1471-2369-15-43.

[5] M. Caldeira *et al.*, “Allergic asthma exhaled breath metabolome: A challenge for comprehensive two-dimensional gas chromatography,” *J Chromatogr A*, vol. 1254, pp. 87–97, Sep. 2012, doi: 10.1016/j.chroma.2012.07.023.

[6] M. Koureas, P. Kirgou, G. Amoutzias, C. Hadjichristodoulou, K. Gourgoulianis, and A. Tsakalof, “Target analysis of volatile organic compounds in exhaled breath for lung cancer discrimination from other pulmonary diseases and healthy persons,” *Metabolites*, vol. 10, no. 8, pp. 1–18, Aug. 2020, doi: 10.3390/metabo10080317.

[7] K. Murtada, V. Galpin, J. J. Grandy, V. Singh, F. Sanchez, and J. Pawliszyn, “Development of porous carbon/polydimethylsiloxane thin-film solid-phase microextraction membranes to facilitate on-site sampling of volatile organic compounds,” *Sustain Chem Pharm*, vol. 21, Jun. 2021, doi: 10.1016/j.scp.2021.100435.

[8] M. Phillips *et al.*, “Prediction of breast cancer risk with volatile biomarkers in breath,” *Breast Cancer Res Treat*, vol. 170, no. 2, pp. 343–350, Jul. 2018, doi: 10.1007/s10549-018-4764-4.

[9] P. Brinkman *et al.*, “Exhaled breath profiles in the monitoring of loss of control and clinical recovery in asthma,” *Clinical and Experimental Allergy*, vol. 47, no. 9, pp. 1159–1169, Sep. 2017, doi: 10.1111/cea.12965.

[10] Y. Y. Broza *et al.*, “Exhaled Breath Markers for Nonimaging and Noninvasive Measures for Detection of Multiple Sclerosis,” *ACS Chem Neurosci*, vol. 8, no. 11, pp. 2402–2413, Nov. 2017, doi: 10.1021/acschemneuro.7b00181.

[11] A. Z. Berna *et al.*, “Reproducible Breath Metabolite Changes in Children with SARS-CoV-2 Infection,” *ACS Infect Dis*, vol. 7, no. 9, pp. 2596–2603, Sep. 2021, doi: 10.1021/acsinfecdis.1c00248.

[12] E. Smirnova *et al.*, “Predictive performance of selected breath volatile organic carbon compounds in stage 1 lung cancer,” *Transl Lung Cancer Res*, vol. 11, no. 6, pp. 1009–1018, Jun. 2022, doi: 10.21037/tlcr-21-953.
