# Supplementary material for: Unraveling the potential of breath and sweat VOC capture devices for human disease detection: a systematic-like review of canine olfaction and GC-MS analysis
Source: Front Chem. 2023 Nov 1;11:1282450. doi: 10.3389/fchem.2023.1282450 (PMC10646374; doi:10.3389/fchem.2023.1282450)
Supplement: Supplementary file 3 [file Table3.DOCX]

**Table 3: Comparison of different odor capture systems suitable for both canine olfaction and GC-MS analysis**

| **Sampling device** | **Sampling source** | **Dog compatibility** | **score** | **Pollution emitted by the device** | **score** | **Composition** | **score** | **GC-MS injection** | **score** | **Device storage/VOC preservation** | **score** | **Sum/10** | **Reference** |
| --- | --- | --- | --- | --- | --- | --- | --- | --- | --- | --- | --- | --- | --- |
| **Getxent^®^ tubes** | sweat | yes | 2 | ready-to-use | 2 | Patented polymer, adsorption of VOC | 2 | extraction step required** (possible loss of VOC) | 1 | stored up to 6 months at -20°C [supplier sources] | 2 | **9** | [1], [2] |
| **Sorbstar^®^** | sweat | yes | 2 | ready-to-use | 2 | Patented polymer, adsorption of VOC | 2 | direct TD injection | 2 | stored at 4°C, storage time is unknown | 0 | **8** | [3]–[5] |
| **PDMS-based patch** (Sorptive tape extraction) | sweat | yes (presented to dogs in cleaned* metal cans [21]-[23]) | 2 | need to be conditioned | 1 | PDMS, adsorption of VOC | 2 | direct TD injection | 2 | stored at 4 °C for no more than 24h [108], no more than 72h [107] | 1 | **8** | [6]–[9] |
| **Twister** (SBSE) | sweat | conceivable (similar size as the Sorbstar^®^ tube) | 1 | need to be conditioned (can be reused) | 1 | PDMS, adsorption of VOC | 2 | direct TD injection | 2 | stored at 4°C, no longer than 14 days [supplier sources] | 1 | **7** | sweat sampling [10] |
| **PDMS tube** (wristband) | sweat | conceivable (easy to handle, size similar to Getxent**^®^** tubes) | 1 | need to be conditioned | 1 | PDMS, adsorption of VOC | 2 | direct TD injection | 2 | stored in aluminum foil at 4 °C for no more than 48 h before analysis [106] | 1 | **7** | [11] |
| **Gauzes** (in direct contact with the skin or sampled with STU-100 tool used in forensic) | sweat | yes | 2 | need to be conditioned | 1 | cellulose fibers (cotton), absorption of VOC | 1 | extraction step required** (possible loss of VOC) | 1 | gauzes stored at -20°C up to 6 months [46], gauzes stored in resealable polyethylene bags for two weeks [54] | 2 | **7** | [12]–[14], related to STU100 [15]–[17] |
| **Chirurgical face masks** | Breath, Exhaled Breath Condensate | yes | 2 | need to be conditioned | 1 | cellulose fibers (cotton), absorption of VOC | 1 | extraction step required** (possible loss of VOC) | 1 | face masks stored at 4°C no longer than a week [45] | 1 | **6** | [18], [19] |
| **Clothes (T-shirt or socks)** | sweat | yes | 2 | need to be washed with a neutral soak | 1 | cellulose fibers (cotton), absorption of VOC | 2 | extraction step required** (possible loss of VOC) | 1 | stored at -12°C, storage time is unknown [115] | 0 | **6** | T-shirt [20], [21], socks [22] |

* Cleaned with pentane and subsequently baked at 150◦C for 2h.

** Extraction can be carried out by solvent, static HS, DHS or SPME

The rating criteria are the following:

1. **Dog compatibility**. If the device is already used in forensic or in another context as an aid for detection dogs training, the score is 2/2. If the device has not been tested with dogs yet, it provides a score of 1/2.
2. **Pollution/VOC emitted by the device itself**. To eliminate VOC released by the device itself, materials must be conditioned before use. If the device is already clean and ready-to-use, the score obtained is 2/2. If it needs to be conditioned before use, 1/2 is attributed.
3. **Composition.** If the device consists of an absorbent support (like a cotton gauze or a T-shirt), the score of 1/2 is given. If the material is made of a particular polymer commonly used in analytical chemistry and which allows the adsorption/desorption of VOC, a score of 2/2 is attributed.
4. **GC-MS injection**. If the thermal desorption (TD injection) is possible, the score of 2/2 is attributed. If an extraction step is necessary, the score of 1/2 is given.
5. **Device storage/VOC preservation**. The score of 2/2 is attributed for “long time” storage (>14 days). For a shorter storage time (<14 days), the score of 1/2 is given. No recommendations found in the literature gives 0/2.

References

[1] D. Grandjean *et al.*, “Can the detection dog alert on COVID-19 positive persons by sniffing axillary sweat samples? A proof-of-concept study,” *PLoS One*, vol. 15, no. 12 December, Dec. 2020, doi: 10.1371/journal.pone.0243122.

[2] D. Grandjean *et al.*, “Identifying SARS-COV-2 infected patients through canine olfactive detection on axillary sweat samples; study of observed sensitivities and specificities within a group of trained dogs,” *PLoS One*, vol. 17, no. 2, Feb. 2022, doi: 10.1371/journal.pone.0262631.

[3] V. Cuzuel, “Développement d’une stratégie de caractérisation chimique de la signature odorante d’individus par l’analyse chimiométrique de données issues de méthodes séparatives multidimensionnelles.” [Online]. Available: https://tel.archives-ouvertes.fr/tel-01680821v2

[4] V. Cuzuel *et al.*, “Origin, Analytical Characterization, and Use of Human Odor in Forensics,” *J Forensic Sci*, vol. 62, no. 2, pp. 330–350, Mar. 2017, doi: 10.1111/1556-4029.13394.

[5] V. Cuzuel *et al.*, “Sampling method development and optimization in view of human hand odor analysis by thermal desorption coupled with gas chromatography and mass spectrometry,” *Anal Bioanal Chem*, vol. 409, no. 21, pp. 5113–5124, Aug. 2017, doi: 10.1007/s00216-017-0458-8.

[6] R. Jiang, E. Cudjoe, B. Bojko, T. Abaffy, and J. Pawliszyn, “A non-invasive method for in vivo skin volatile compounds sampling,” *Anal Chim Acta*, vol. 804, pp. 111–119, Dec. 2013, doi: 10.1016/j.aca.2013.09.056.

[7] W. A. MacCrehan, M. Young, and M. M. Schantz, “Measurements of vapor capture-and-release behavior of PDMS-based canine training aids for explosive odorants,” *Forensic Chemistry*, vol. 11, pp. 58–64, Dec. 2018, doi: 10.1016/j.forc.2018.09.002.

[8] W. MacCrehan, M. Young, M. Schantz, T. Craig Angle, P. Waggoner, and T. Fischer, “Two-temperature preparation method for PDMS-based canine training aids for explosives,” *Forensic Chemistry*, vol. 21, Dec. 2020, doi: 10.1016/j.forc.2020.100290.

[9] A. G. Simon, K. Van Arsdale, J. Barrow, and J. Wagner, “Real-time monitoring of TATP released from PDMS-based canine training aids versus bulk TATP using DART-MS,” *Forensic Chemistry*, vol. 23, May 2021, doi: 10.1016/j.forc.2021.100315.

[10] H. A. Soini, K. E. Bruce, I. Klouckova, R. G. Brereton, D. J. Penn, and M. V. Novotny, “In situ surface sampling of biological objects and preconcentration of their volatiles for chromatographic analysis,” *Anal Chem*, vol. 78, no. 20, pp. 7161–7168, Oct. 2006, doi: 10.1021/ac0606204.

[11] M. Wooding, E. R. Rohwer, and Y. Naudé, “Non-invasive sorptive extraction for the separation of human skin surface chemicals using comprehensive gas chromatography coupled to time-of-flight mass spectrometry: A mosquito-host biting site investigation,” *J Sep Sci*, vol. 43, no. 22, pp. 4202–4215, Nov. 2020, doi: 10.1002/jssc.202000522.

[12] E. Sinclair *et al.*, “Validating differential volatilome profiles in Parkinson’s disease,” *ACS Cent Sci*, vol. 7, no. 2, pp. 300–306, Feb. 2021, doi: 10.1021/acscentsci.0c01028.

[13] D. K. Trivedi *et al.*, “Discovery of Volatile Biomarkers of Parkinson’s Disease from Sebum,” *ACS Cent Sci*, vol. 5, no. 4, pp. 599–606, Apr. 2019, doi: 10.1021/acscentsci.8b00879.

[14] A. M. Curran, P. A. Prada, and K. G. Furton, “The differentiation of the volatile organic signatures of individuals through SPME-GC/ms of characteristic human scent compounds,” *J Forensic Sci*, vol. 55, no. 1, pp. 50–57, Jan. 2010, doi: 10.1111/j.1556-4029.2009.01236.x.

[15] R. A. Stockham, D. L. Slavin, and B. Handler, “Specialized Use of Human Scent in Criminal Investigations.”

[16] A. M. Curran, P. A. Prada, and K. G. Furton, “Canine human scent identifications with post-blast debris collected from improvised explosive devices,” *Forensic Sci Int*, vol. 199, no. 1–3, pp. 103–108, Jun. 2010, doi: 10.1016/j.forsciint.2010.03.021.

[17] L. E. Degreeff and K. G. Furton, “Collection and identification of human remains volatiles by non-contact, dynamic airflow sampling and SPME-GC/MS using various sorbent materials,” *Anal Bioanal Chem*, vol. 401, no. 4, pp. 1295–1307, Sep. 2011, doi: 10.1007/s00216-011-5167-0.

[18] P. Devillier *et al.*, “Biomedical detection dogs for the identification of SARS-CoV-2 infections from axillary sweat and breath samples∗∗,” *J Breath Res*, vol. 16, no. 3, Jul. 2022, doi: 10.1088/1752-7163/ac5d8c.

[19] J. Mendel *et al.*, “Preliminary accuracy of COVID-19 odor detection by canines and HS-SPME-GC-MS using exhaled breath samples,” *Forensic Sci Int*, vol. 3, Jan. 2021, doi: 10.1016/j.fsisyn.2021.100155.

[20] S. Haze *et al.*, “2-Nonenal Newly Found in Human Body Odor Tends to Increase with Aging.”

[21] M. Gallagher, C. J. Wysocki, J. J. Leyden, A. I. Spielman, X. Sun, and G. Preti, “Analyses of volatile organic compounds from human skin,” *British Journal of Dermatology*, vol. 159, no. 4, pp. 780–791, Oct. 2008, doi: 10.1111/j.1365-2133.2008.08748.x.

[22] C. Guest *et al.*, “Trained dogs identify people with malaria parasites by their odour,” *The Lancet Infectious Diseases*, vol. 19, no. 6. Lancet Publishing Group, pp. 578–580, Jun. 01, 2019. doi: 10.1016/S1473-3099(19)30220-8.
